# Empiric intralesional tumescent drug delivery of antimicrobials effectively treated a painful necrotizing skin infection

**DOI:** 10.1016/j.jdcr.2024.03.008

**Published:** 2024-03-26

**Authors:** Paytra A. Klein, Gerald A. Wagner, Ronald J. Barr, Jeffrey A. Klein, Roy S. Rogers

**Affiliations:** a Albany Medical College, Albany, New York; b Department of Pediatrics, University of California Irvine, Irvine, California; c Department of Dermatology, University of California Irvine, Irvine, California; d Department of Dermatology, Mayo Clinic Arizona, Phoenix, Arizona

**Keywords:** amikacin, amphotericin B, antibiotic, antimicrobial, cefazolin, drug delivery, ecthyma gangrenosum, empiric treatment, epinephrine, infection, infiltration, injection, intralesional, leukemia cutis, lidocaine, necrosis, necrotic, necrotizing, oral dermatology, polymicrobial, skin infection, targeted, TDD, TEL, tumescent antibiotic delivery, tumescent delivery, tumescent drug delivery, tumescent epinephrine, tumescent epinephrine lidocaine, tumescent infiltration, tumescent injection, tumescent lidocaine, tumescent local anesthesia, ulcer

## Introduction

Tumescent epinephrine lidocaine (TEL) solutions consist of dilute epinephrine (≤1 mg/L) and lidocaine (≤1gm/L).^1^ TEL is approximately a 10-fold dilution of 1% lidocaine with epinephrine 1:100,000.^1^ Relatively large volumes of subcutaneous TEL produce tumescent swelling and firmness. When a lidocaine-epinephrine solution is both diluted and slowly injected using hypodermic needles, there is little pain associated with its injection; rendering the addition of bicarbonate to the solution unnecessary.

Tumescent drug delivery (TDD) is defined as a subcutaneous infiltration of drugs dissolved in a TEL solution. Many localized lesions are optimally treated by targeted local drug delivery. TDD is a novel mode of targeted drug delivery for localized lesions with unprecedented pharmacokinetic and pharmacodynamic properties that are unmatched by intravenous (IV), intramuscular, or per os/oral delivery. We report a case of an immunocompromised patient with an exquisitely painful rapidly enlarging necrotizing ulcer of the lower lip and oral commissure that failed systemic antibiotic therapy. The lesion rapidly improved following empiric treatment with intralesional infiltration of a TDD solution consisting of dilute epinephrine, lidocaine, amikacin, cefazolin, and amphotericin-B.

## Case report

**Fig 1:**
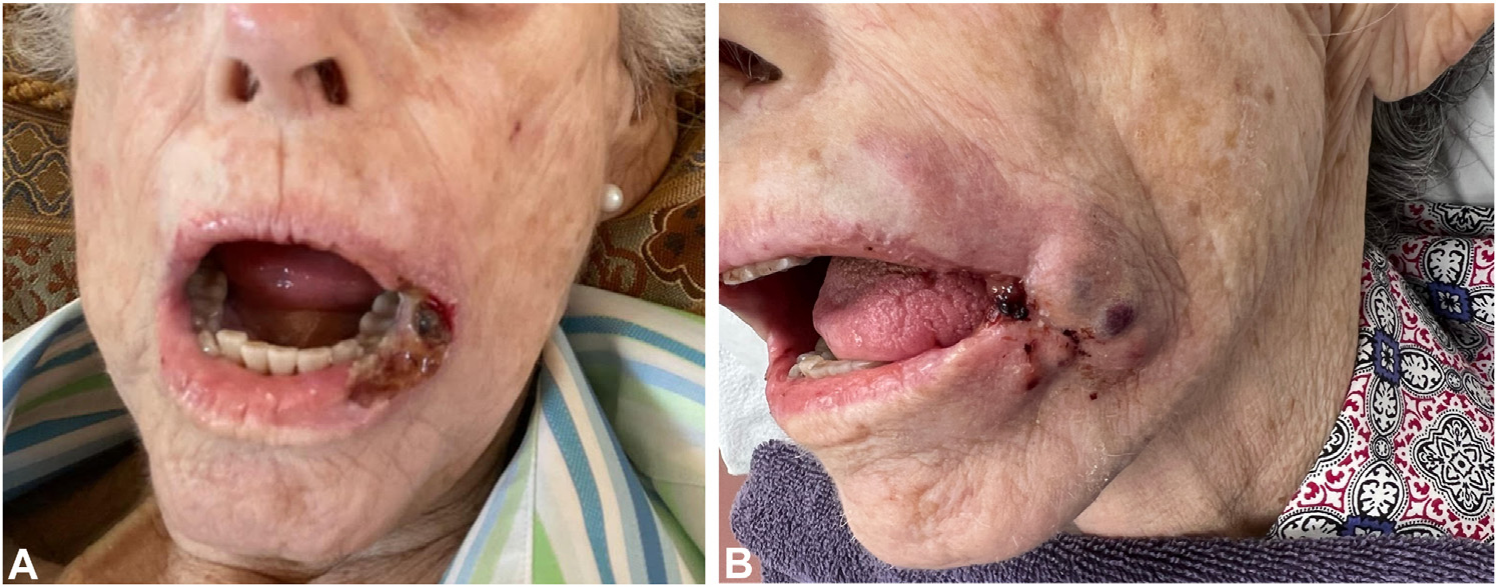
Ulceration before-and-after: **A,** Before tumescent drug delivery (TDD) of antimicrobials. **B,** 10 days after TDD of antimicrobials – ulceration was a third of its original size.

An 81-year-old female with myelodysplastic syndrome with excessive blasts-2 (MDS-EB2) presented with an extremely painful 1 × 2.2 cm ulceration of the left lower lip and oral commissure (Fig 1, *A*). The pain was incapacitating and prevented the patient from opening her mouth to eat. She was not septic. There was no remote history of herpes simplex or zoster infections. The ulceration and pain had progressively worsened despite receiving systemic chemotherapy, blood transfusions, IV antimicrobials, and opioids from her oncologists. The patient then presented to our dermatology practice. While awaiting initial culture and punch biopsy results, the patient was treated empirically with 2 subcutaneous intralesional injections, first with 15 ml and the next day with 45 ml, of a dilute tumescent solution consisting of epinephrine (1 mg/L), lidocaine (1gm/L), amikacin (1gm/L; for gram-negatives), cefazolin (2gm/L; for gram-positives), and amphotericin-B (100 mg/L; for fungi). Pain ceased immediately and never returned.

**Fig 2:**
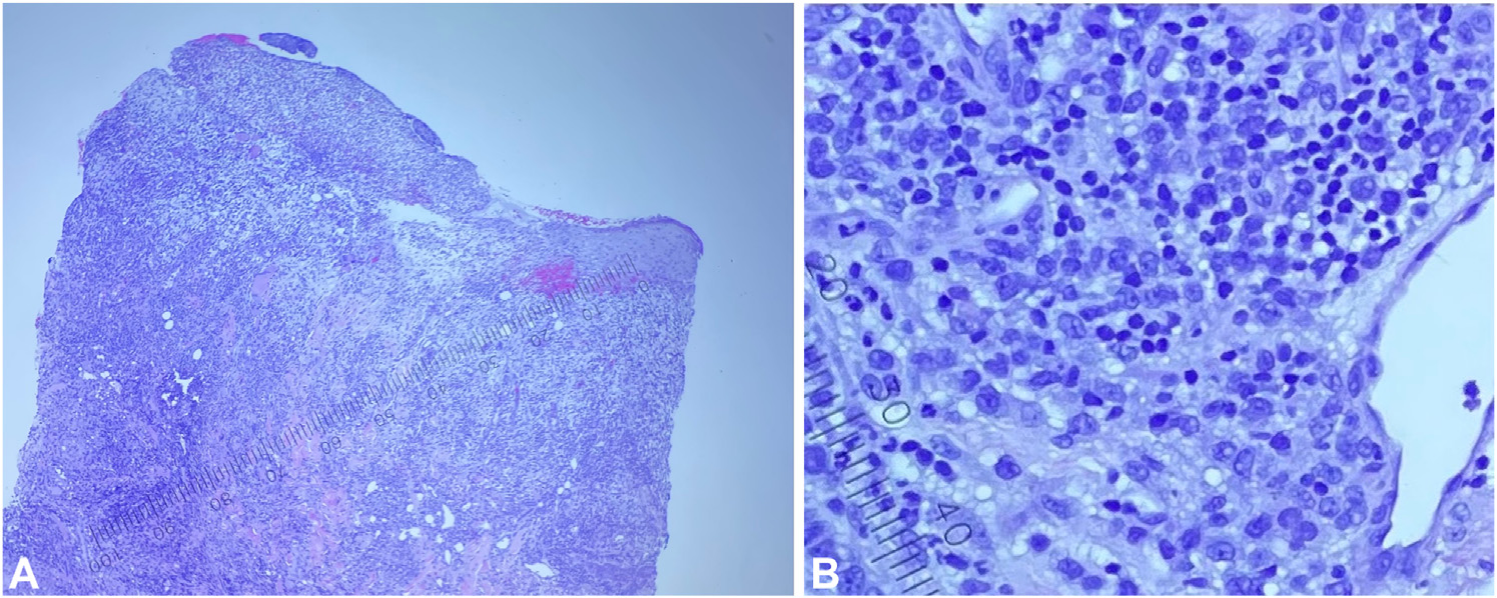
**A** and **B,** Oral commissure ulceration punch biopsy (hematoxylin and eosin [H&E], 100×-400×): Dense diffuse dermal infiltrate consists of myeloblasts with an inflammatory background of lymphocytes and scattered neutrophils.

Culture swabs eventually revealed Enterococcus haemolyticus and Staphylococcus haemolyticus. Punch biopsy revealed leukemia cutis (LC) in the deeper portion of the specimen and superficial reactive neutrophils (Figs 2, *A* and *B*). Histochemical stains revealed that some cells were positive for CD33 (expressed by myeloid stem cells).

Within 10 days, the ulcer was one-third of its original size (Fig 1, *B*). On day 34, the patient died without oral pain and the ulceration had healed completely. Postmortem diagnosis was ecthyma gangrenosum (EG) with underlying LC.

## Discussion

LC is the infiltration of neoplastic leukocytes into the skin that presents in myelodysplastic or leukemic patients as violaceous cutaneous lesions which may ulcerate.^2^ EG is a cutaneous infection that manifests in immunocompromised patients as erythematous papules that develop into pustules, bullae, or crusted, necrotic, gangrenous ulcers with eschar surrounded by raised erythematous borders.^3^ Although EG is often a cutaneous manifestation of pseudomonas bacteremia, microbes can directly inoculate existing lesions of nonseptic patients and cause localized EG.^4^ Standard treatment options for EG and LC include aggressive systemic delivery of antimicrobials and chemotherapy, respectively.^2^^,^^3^ This patient received both IV antimicrobials and systemic chemotherapy, however the ulcer continued to enlarge and became increasingly painful. The use of systemic antimicrobial delivery to treat localized necrotic skin infections may be insufficient because of capillary necrosis, interstitial edema, reduced intralesional vascular perfusion, and impaired local antimicrobial bioavailability. Targeted intralesional antimicrobial injection reduces total antimicrobial dosage, reduces the risk of systemic toxicity, and is consistent with responsible antibiotic stewardship.

There are 3 requirements for a safe and effective tumescent interstitial injection of a drug. The interstitial drug concentration must be: (1) above the threshold for therapeutic efficacy, (2) below the threshold for local tissue toxicity, and (3) above the intralesional drug concentration that can be achieved by IV delivery.

The formulation of a safe TDD solution is an art that requires a knowledge of tumescent pharmacokinetics and the pharmacology of component drugs and their drug-drug interactions. An initial estimate for the concentration of any drug (D) in a TDD solution requires a literature search for a reported peak serum concentration (Cmax) of D following a single IV injection. In our experience, 10 × Cmax is a reasonable and conservative first estimate of a safe and effective concentration of D in a TDD solution.

Amikacin has a reported Cmax = 80 mg/L.^5^ We chose 1 gm/L = 12 × Cmax of amikacin for the TDD solution because 12 × Cmax is clinically indistinguishable from 10 × Cmax and because the use of (12 × Cmax) = (12 × 80 mg/L) ≈ 1gm/L simplifies the actual task of mixing of the TDD solution and reduces the risk of an error. Cefazolin at 2 gm/L in a TDD solution is known to be safe and effective.^6^ Liposomal amphotericin-B has a mean serum Cmax 3.5 mg/L.^7^^,^^8^ We chose 100 mg/L for our TDD solution. Although the extravasation of highly concentrated IV-administered amphotericin-B into surrounding extravascular tissues has been reported to cause tissue irritation and damage,^9^ the extreme dilution of amphotericin-B in a TDD solution lessens the risk of tissue toxicity. A TDD solution ought to be discarded if there is any visual evidence of a precipitate. The safety of a novel TDD formulation can be experienced by a self-injection a small aliquot of the solution.

The maximum safe dosage of tumescent lidocaine is 28 mg/kg for healthy adults and 21 mg/kg for patients who are very thin, frail, or elderly.^1^ Therefore, in a 50 kg person, 21 mg/kg of lidocaine in a 1gm/L solution permits 1000 ml of TEL.^1^

The actual injection of a TDD solution requires a gentle technique using a gradually increasing sequence of hypodermic needles (eg, 30 g × 4 mm, 25 g × 25 mm, 22 g × 25 mm, 20 g × 37 mm). Small TDD volumes can be infiltrated using individual syringes. Significantly larger volumes of a TDD solutions require a high precision, digitally controlled peristaltic roller pump with accurate fluid flowrate selection in increments of 1 rotation per minute (RPM), equivalent to 1.7 ml/min. For painless TDD, the pump RPM typically ranges from 5RPM to 60RPM depending on the hypodermic needle size and the anatomic target of the TDD. It useful to note that the interstitial concentration of drug **D** immediately after TDD is virtually equal to the concentration of **D** in the TDD solution.

For cutaneous targets, the pharmacokinetic advantages of TDD are far superior to those of IV, intramuscular, or per os/oral drug delivery. Years of clinical experience providing tumescent antibiotic delivery for dermatologic surgery has shown that TEL is an ideal excipient (carrier) solution for interstitial antimicrobial injections. The dilute epinephrine in TEL subcutaneous infiltration produces capillary vasoconstriction, delays systemic absorption of drugs in a TEL solution, prolongs local drug effect, and increases localized drug bioavailability.^10^^,^^11^ The large volume provides a significant reservoir of TDD solution which complements the epinephrine-induced capillary vasoconstriction. Lidocaine is bactericidal and anti-inflammatory.^12^, ^13^, ^14^, ^15^ Importantly, lidocaine eliminates any pain associated the injection of other drugs. Subcutaneous TDD increases the local concentration of drugs, reduces the total milligram dose, and reduces the risk of adverse systemic effects associated with larger IV drug doses.

While there is evidence supporting the subcutaneous administration of antibiotics,^16^ it is underutilized.^17^ Future research is warranted to explore the potential uses of TDD for the targeted treatment of serious and challenging dermatologic infections such as mycetoma (bacterial actinomycetoma and fungal eumycetoma),^18^ diabetic foot ulcers,^19^^,^^20^ cutaneous leishmaniasis,^21^^,^^22^ necrotizing faciitis,^23^ and the pre-emptive treatment use of TDD to reduce the risk of surgical site infections.^6^^,^^24^

In conclusion, we report the successful empiric treatment of an exquisitely painful necrotic ulceration, clinically consistent with EG, using targeted intralesional TDD of epinephrine, lidocaine (for pain control), amikacin (for gram-negatives), cefazolin (for gram-positives), and amphotericin-B (for fungi). To our knowledge, this is the first reported case using TDD of epinephrine, lidocaine, and antimicrobials to successfully treat a cutaneous necrotizing ulceration. Further research is needed to support the inclusion of TDD in the treatment of intractable cutaneous infections and the preliminary empiric treatment of extremely painful necrotizing skin lesions.

## Conflicts of interest

Jeffrey A. Klein owns HK Surgical, Inc (a surgical supply and equipment company that develops infiltration pumps that can be used for Tumescent Drug Delivery). Jeffrey A. Klein and Paytra A. Klein have patents regarding Tumescent Drug Delivery.
